# The Role of Voltage-Gated Sodium Channel 1.8 in the Effect of Atropine on Heart Rate: Evidence From a Retrospective Clinical Study and Mouse Model

**DOI:** 10.3389/fphar.2020.01163

**Published:** 2020-07-31

**Authors:** Baowen Liu, Ningbo Li, Jin Zhang, Yi Liu, Mi Zhang, Yishun Hong, Wenyao Wu, Xianwei Zhang, Guangyou Duan

**Affiliations:** ^1^Department of Anesthesiology, The Second Affiliated Hospital, Chongqing Medical University, Chongqing, China; ^2^Department of Anesthesiology, Tongji Hospital, Tongji Medical College, Huazhong University of Science and Technology, Wuhan, China

**Keywords:** *SCN10A*/Na_V_1.8, rs6795970, atropine, heart rate, methoctramine

## Abstract

Atropine is commonly used to counter the effects of the parasympathetic neurotransmitter acetylcholine on heart rate in clinical practice, such as in the perioperative period; however, individual differences in the response to atropine are huge. The association between *SCN10A*/voltage-gated sodium channel 1.8 (Na_V_1.8) and cardiac conduction has been demonstrated; however, the exact role of *SCN10A*/Na_V_1.8 in the heart rate response to atropine remains unclear. To identify the role of *SCN10A* variants that influence the heart rate responses to atropine, we carried out a retrospective study in 1,005 Han Chinese subjects. Our results showed that rs6795970 was associated with the heart rate response to atropine. The heart rate responses to atropine and methoctramine in Na_V_1.8 knockout mice were lower, whereas the heart rate response to isoproterenol was like those in wild type mice. Furthermore, we observed that the Na_V_1.8 blocker A-803467 alleviated the heart rate response to atropine in wild type mice. The retrospective study revealed a previously unknown role of Na_V_1.8 in controlling the heart rate response to atropine, as shown by the animal study, a speculative mechanism that may involve the cardiac muscarinic acetylcholine receptor M2.

## Introduction

Atropine is a competitive antagonist of acetylcholine muscarinic receptors because of its similar structure with acetylcholine, a parasympathetic neurotransmitter (Lakstygal et al., 2019). An increase in parasympathetic activity can lead to transient bradycardia and even to asystole, which can affect cerebral oxygenation and perfusion (Rasmussen et al., 2007; Anastasian et al., 2014). As an anticholinergic medication, atropine is commonly used to counter the effects of acetylcholine on heart rate and rapidly increase the heart rate of patients with bradycardia in clinical practice, such as during the perioperative period. However, there is marked interindividual variation in the responses to atropine (Lee et al., 2008); thus, the atropine test is often used to evaluate the sensitivity of the individual to atropine. The mechanism underlying the differences in atropine sensitivity among individuals is unclear. Thus, the exploration of a mechanism will elucidate the individual variation to atropine and may also provide a new target for clinical heart rate intervention.

Recently, several genome-wide association studies have shown that *SCN10A*, which is locus on chromosome 3 and encodes voltage-gated sodium channel 1.8 (Na_V_1.8), is involved in cardiac conduction and dysrhythmia (Chambers et al., 2010; Holm et al., 2010; Pfeufer et al., 2010; Sotoodehnia et al., 2010). Na_V_1.8 is primarily expressed in small and medium diameter nociceptive sensory neurons of the dorsal root ganglia and plays a key role in the transmission of pain perception (Akopian et al., 1999). The Na_V_1.8 gene is adjacent to the gene of the canonical cardiac sodium channel Na_V_1.5 and there is 70.4% sequence similarity between these channels (Chen et al., 2016). The expression of Na_V_1.8 has been shown in mouse and human heart tissue (Chambers et al., 2010). However, the expression of Na_V_1.8 in the heart is controversial. Facer et al. showed that Na_V_1.8 is present in both nerves and cardiomyocytes of the human heart, using immunohistological methods (Facer et al., 2011). However, other studies demonstrated substantial Na_V_1.8 expressed in isolated murine intracardiac ganglia neurons, but no Na_V_1.8 expression in isolated ventricular myocytes (Verkerk et al., 2012; Casini et al., 2019). Furthermore, an increasing number of studies have focused on the association between Na_V_1.8 and cardiac conduction disease. For example, the common variant rs6795970 in *SCN10A* is associated with the heart rate response (Delaney et al., 2014). In addition, several studies have shown that Na_V_1.8 is associated with atrial fibrillation (AF) (Qi et al., 2014; Chen et al., 2016). Moreover, the blockage of Na_V_1.8 by A-803467 increases the PR- and QRS-interval in mice and decreases the incidence of AF in dogs (Sotoodehnia et al., 2010; Chen et al., 2016). These observations suggest that Na_V_1.8 is a critical modifier of cardiac conduction, but its role in the regulation of heart rate needs further study.

This study used retrospective analysis to investigate the roles of variants of Na_V_1.8 in the effects of atropine on heart rate. Additionally, a drug experiment, based on an animal model using Na_V_1.8 knockout mice, was performed to validate the clinical results.

## Material and Methods

### Study Design

This study had two parts. First, a retrospective study was performed to explore the role of *SCN10A* variants in the effects of atropine on heart rate. Second, to explore the mechanism underlying the role of Na_V_1.8 in heart rate, an animal experiment was designed to investigate the effects of atropine, methoctramine, and isoproterenol on heart rate using Na_V_1.8 knock-out mice and wild type mice. All patient data and all experimental data of this study can be acquired from the corresponding author on a reasonable request *via* e-mail.

### Patients and Electrocardiographic Data

**Figure 1:**
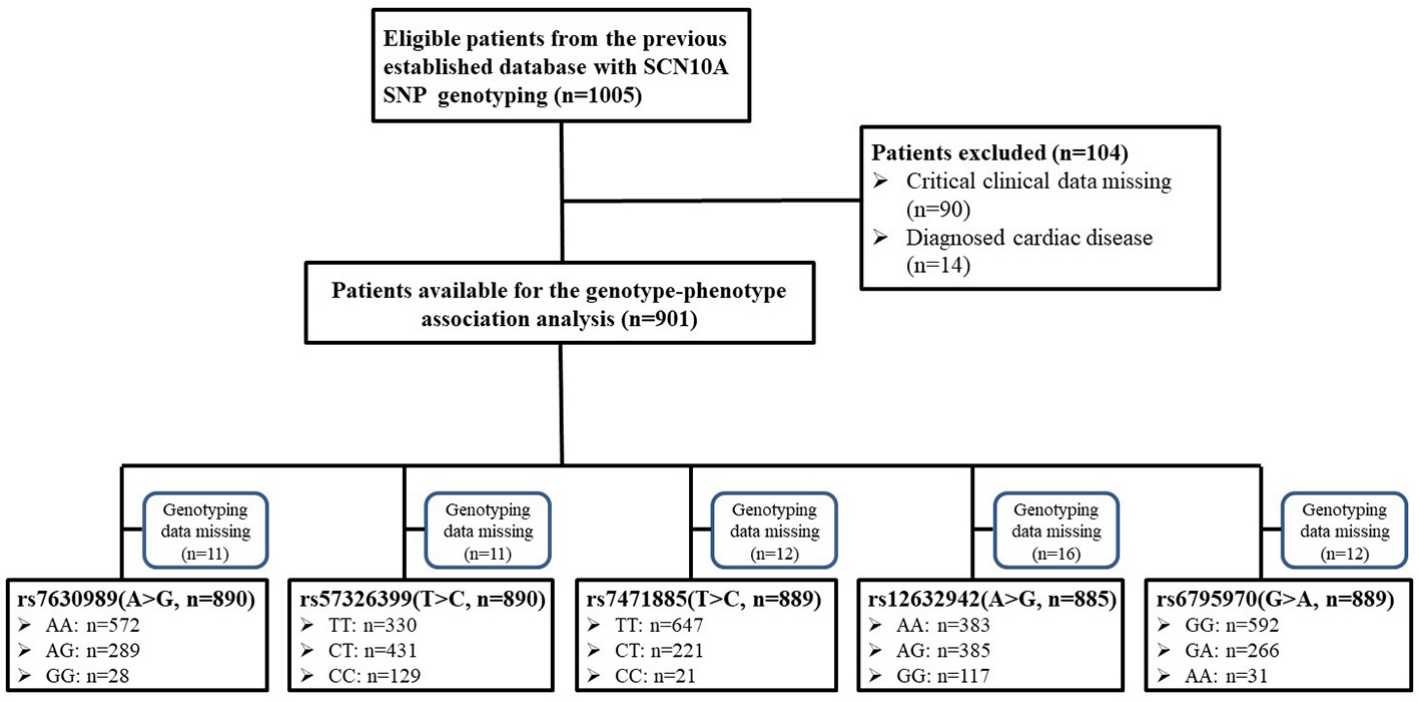
Diagram of patient inclusion summary.

The study was a retrospective study and the protocol was approved by the Institutional Ethics Committee of Tongji Hospital, Tongji Medical College, Huazhong University of Science and Technology. The cohort was obtained from our previous study (Duan et al., 2016a) and, because this study was based on the previous data, written informed consent was waived. A total of 1,005 Han Chinese women, who were scheduled for elective gynecological surgery, were considered for this study (**Figure 1**). The inclusion criteria were as follows: Han Chinese; grouped based on the American Society of Anesthesiologists physical status I–II. The exclusion criteria were as follows: data missing; smoking, alcohol, or drug abuse; known cardiovascular disease; or New York Heart Association class III–IV. An electrocardiographic examination was performed the day before the surgery. Electrocardiographic data, including PR interval, QRS duration, and, QT interval were collected from the electronic medical record system using the patients’ identification number. All patients received preoperative medication. An intramuscular injection of 0.5 mg of atropine was used before the surgery in the operating waiting room. The heart rates before and approximately 30 min after atropine administration were recorded. In addition, the demographic characteristic, such as age and body mass index (BMI), were collected. The systolic blood pressure, diastolic blood pressure, mean arterial pressure (MAP), and the time [morning (06:00–12:00) or afternoon (12:00–18:00)] of atropine administration were recorded.

### Included Single Nucleotide Polymorphisms (SNPs)

A total of 5 *SCN10A* SNPs were included in the analysis. The SNP rs6795970 (nucleotide level: NM_006514.2:c.3218 G>A), whose minor allele causes a non-synonymous substitution (NP_006505.2: protein.Ala1073Val, using the three letter amino acid code), is the most frequently reported non-synonymous *SCN10A* SNP to be associated with cardiac conduction (Behr et al., 2015; Iio et al., 2015; Macri et al., 2018) and was included. Four additional SNPs, rs12632942 (c.3218 A>G, protein.Leu1092Pro), rs7630989 (c.1525 A>G, protein.Ser509Pro), rs57326399 (c.2884 T>C, protein.Ile962Val), and rs74717885 (c.618 T>C, protein.Ile206Met), were selected based on a minor allele frequency ≥5% and their locations in exons, which can cause amino acid substitutions.

### Animals

Na_V_1.8 knockout mice, generated as described previously (Nassar et al., 2004; Stirling et al., 2005), were gifted from Professor Stephen G. Waxman (Yale University School of Medicine, USA) and bred at our facility in Wuhan. Congenic wild-type mice were provided by the Animal Center of Hubei Province, China. Male and female adult mice (20–25 g) were maintained under controlled conditions (temperature: 22–24°C, humidity: 50–60%, light: 12 h light/12 h dark cycle, standard laboratory rodent chow, and water *ad libitum*) in the animal center of Tongji Hospital. Polymerase chain reaction genotyping was performed as described previously (Nassar et al., 2004; Gautron et al., 2011). All experimental protocols were approved by the ethical committee of Tongji Hospital, Tongji Medical College, Huazhong University of Science and Technology (IRB ID : TJ-A20181228) and followed the guidelines outlined by the National Institutes of Health Guide for the Care and Use of Laboratory Animals.

### Drugs

Atropine (1044990, purity ≥99%, Sigma, St. Louis, MO, USA) was dissolved in sterile isotonic saline and was administered by intraperitoneal injection at a dose of 3 mg/kg (Wan et al., 2014). Isoproterenol (420355, purity ≥99%, Sigma) was dissolved in sterile isotonic saline and was administered by intraperitoneal injection at a dose of 2 mg/kg (Sato, 2019). Methoctramine (sc-257709, purity ≥97%, Santa Cruz Biotechnology, Dallas, TX, USA), which was used as an antagonist of the muscarinic acetylcholine receptor M2, was dissolved in sterile isotonic saline and was administered by intraperitoneal injection at a dose of 3 mg/kg (Dobryakova et al., 2018). A-803467 (HY-11079, purity = 98.47%, Med Chem Express, Monmouth Junction, NJ, USA), which was used as a blocker of Na_V_1.8, was dissolved in 5% dimethyl sulfoxide and 95% polyethylene glycol 400 in saline and was administered by intraperitoneal injection at a dose of 25 mg/kg. The dose of A-803467 was based on previous studies and our preliminary experiments (Jarvis et al., 2007; Joshi et al., 2009).

### Heart Rate Recording

After intraperitoneal anesthesia with chloral hydrate at a dose of 400 mg/kg, the mice were laid on the back with the right thigh shaved (Cao et al., 2018). A heating pad was used to maintain body temperature. The heart rate was recorded by a MouseOx Pulse Oximeter system (Starr Life Science, Oakmont, PA, USA), using an oximeter probe placed on the thigh of mouse, according to the manufacturer’s instructions (Li et al., 2010).

### Statistical Analysis

The data of the retrospective study are presented as mean ± standard deviation or number (percentage). The SPSS Statistics Version 22.0 statistical package (SPSS Statistics, Inc., Chicago, IL, USA) was used to analyze the data. For the gene association analysis, the sample was tested for each SNP to determine whether the null hypothesis of the Hardy-Weinberg equilibrium (HWE) could be rejected by applying the chi-square method. As the included population was considered as an exploratory cohort, we did not calculate the required sample size before the study. Independent-sample t-test and one-way analysis of variance (ANOVA) test were used to compare the quantitative data between different genotype groups and a chi-square test was performed for qualitative data. A two-way repeated ANOVA with post-hoc paired-samples t-test was used to compare the difference in heart rate before and after atropine use between different genotype groups. In addition, a propensity score-matched analysis was performed to further compare the heart rate after atropine use. The basal characteristics, including age, BMI, MAP, time period, basal heart rate, and electrocardiographic data (including PR interval, QRS duration, and QT interval), were considered as matched factors and matching using a 1:1 nearest neighbor method without re-placement under a logit model was performed.

The data of the animal experiment are presented as mean ± standard error of the mean. The Prism 7.0 Software (GraphPad, San Diego, CA, USA) was used to analyze the data. A two-way repeated ANOVA with a post-hoc paired-samples t-test was used to compare the difference in heart rate between groups. A p-value of <0.05 was considered statistically significant.

## Results

**Figure 2:**
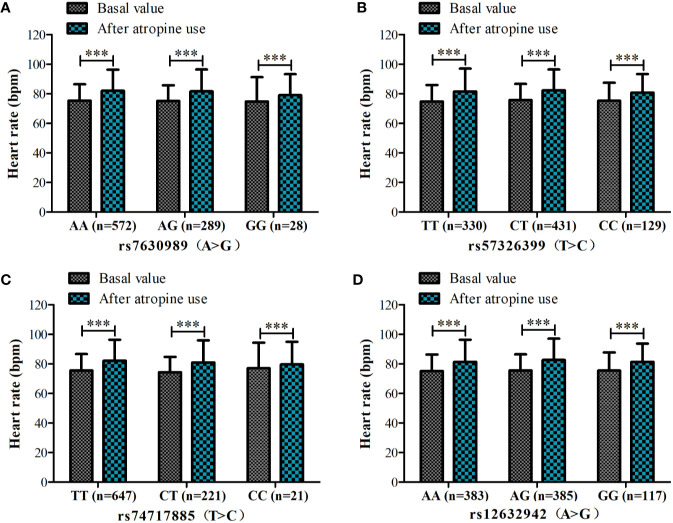
Heart rate before and after atropine use for patients with different genotypes of rs7630989 **(A)**, rs57326399 **(B)**, rs74717885 **(C)**, and rs12632942 **(D)**. ***P < 0.001 compared to basal value.

Because of missing electrocardiographic data and cardiac disease for some subjects, 904 patients were successfully documented and considered for the gene association analysis (**Figure 1**). No significant associations were observed in the heart rate after atropine use among the different genotypes of rs7630989, rs57326399, rs74717885, and rs12632942. Furthermore, for all patients with these genotypes, the heart rate after atropine use was significantly increased compared to the basal value (all *P* < 0.001, **Figure 2**).

**Figure 3:**
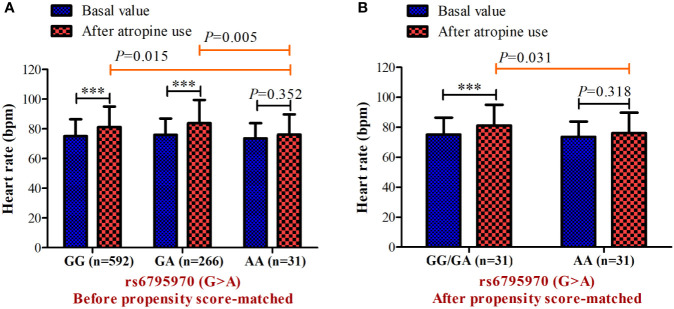
Heart rate before and after atropine use for patients with different genotypes of s6795970 in non-matched cohort **(A)** and matched cohort **(B)**. ***P < 0.001 compared to basal value.

In the association analysis, only the rs6795970 genotype was correlated with heart rate after atropine use (*P* = 0.005). For rs6795970, the HWE value was *P* = 0.852. No significant difference was observed in basal characteristics or other electrophysiological data from patients with different *SCN10A* rs6795970 alleles (**Table 1**). The basal heart rate before the intramuscular injection atropine in patients with different rs6795970 alleles was comparable, but after atropine use the heart rate in patients with AA was significantly lower than those in patients with GG (76.2 ± 13.6 *vs.* 81.4 ± 13.8, *P* = 0.015) and GA (76.2 ± 13.6 *vs.* 83.8 ± 13.9, *P* = 0.005). In patients with GG (*P* < 0.001) and GA, the heart rate after atropine use was significantly increased compared to the basal value (all *P* < 0.001, **Figure 3A**). However, in patients with AA, no significant difference was observed in the heart rate between before and after atropine use (*P* = 0.352). After propensity score matching, patients with AA had a lower heart rate after atropine use than patients with other rs6795970 genotypes (76.2 ± 13.6 *vs.* 84.4 ± 13.8, *P* = 0.032, **Table 2**). No significant difference was observed in the heart rate between before and after atropine use in patients with AA (*P* = 0.318, **Figure 3B**).

**Table 1:** Basal characteristic and electrocardiographic data with different *SCN10A* rs6795970 alleles.

	GG (n=592)	GA (n=266)	AA (n=31)	ANOVA *P*
Age (years)	38.3 ± 10.8	38.7 ± 10.9	40.6 ± 11.2	0.465
BMI (kg/m^2^)	22.3 ± 3.5	22.1 ± 3.4	23.1 ± 3.4	0.210
SBP (mmHg)	116.9 ± 16.4	115.0 ± 17.1	115.1 ± 13.2	0.265
DBP (mmHg)	69.5 ± 11.3	70.3 ± 10.9	68.7 ± 10.1	0.541
MAP (mmHg)	85.1 ± 12.1	85.2 ± 11.3	84.1 ± 10.5	0.895
SPO_2_ (%)	99.1 ± 1.6	99.0 ± 1.5	99.1 ± 1.2	0.741
Atropine use at morning (n[%])	502(84.8%)	220(82.7%)	26(83.9%)	0.740
Heart rate before atropine use (bpm)	75.1 ± 11.3	75.9 ± 10.9	73.4 ± 10.3	0.443
PR interval (ms)	142.9 ± 17.8	141.4 ± 18.6	149.0 ± 20.0^#^	0.085
QRS duration (ms)	83.6 ± 9.5	83.4 ± 10.1	86.1 ± 15.2	0.385
QT interval (ms)	383.6 ± 30.6	383.2 ± 26.9^*^	396.2 ± 27.3^#^	0.072
Heart rate after atropine use (bpm)	81.4 ± 13.8	83.8 ± 15.7^*^	76.2 ± 13.6^##^	0.005

ANOVA, one-way analysis of variance; BMI, body max index; SBP, Systolic blood pressure; DBP, diastolic blood pressure; MAP, mean arterial pressure; ^*^P < 0.05 compared to GG; ^#^P < 0.05 compared to GA; ^##^P < 0.01 compared to GA.

**Table 2:** Basal characteristic and electrocardiographic data with different *SCN10A* rs6795970 alleles after propensity score-matched.

	GG/GA (n=31)	AA (n=31)	Standardized difference	*P* value
Age (years)	39.2 ± 10.3	40.6 ± 11.2	0.132	0.605
BMI (kg/m^2^)	23.9 ± 3.7	23.1 ± 3.4	0.216	0.397
SBP (mmHg)	113.9 ± 11.2	115.1 ± 13.2	0.103	0.686
DBP (mmHg)	70.6 ± 9.0	68.7 ± 10.1	0.205	0.422
MAP (mmHg)	85.0 ± 8.7	84.1 ± 10.5	0.092	0.716
Atropine use at morning (n[%])	29(93.5%)	26(83.9%)	0.309	0.422
Heart rate before atropine use (bpm)	75.9 ± 12.6	73.4 ± 10.3	0.199	0.436
PR interval (ms)	148.4 ± 20.8	149.0 ± 20.0	0.028	0.916
QRS duration (ms)	87.3 ± 10.6	86.1 ± 15.2	0.082	0.727
QT interval (ms)	394.0 ± 26.7	396.2 ± 27.3	0.080	0.756
Heart rate after atropine use (bpm)	84.4 ± 13.8	76.2 ± 13.6	0.559	0.032

BMI, body max index; SBP, Systolic blood pressure; DBP, diastolic blood pressure; MAP, mean arterial pressure.

**Figure 4:**
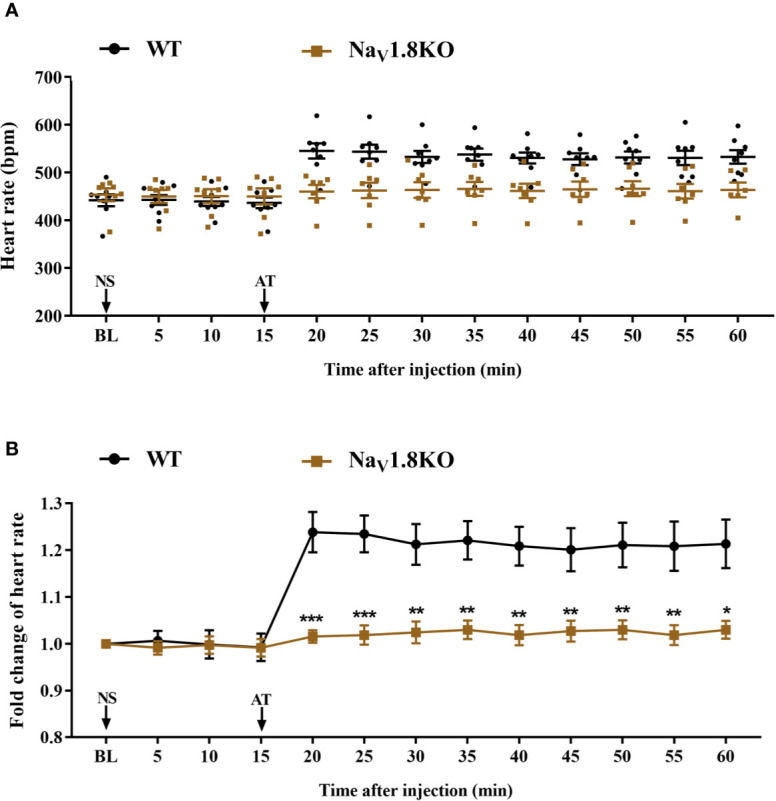
The heart rate response of atropine in Na_V_1.8 knockout mice. **(A)** Scatter diagram of heart rate after atropine treatment. **(B)** Line diagram of fold change of heart rate after atropine treatment. Heart rate was normalized to the value of baseline heart rate for each group. *p < 0.05, **p < 0.01, ***p <0.001 vs wild type mice. WT, wild type; KO, knock out; NS, normal saline; AT, atropine; BL, baseline.

To further explore the correlation between Na_V_1.8 and heart rate response to atropine, we examined the heart rate before and after atropine treatment in Na_V_1.8 knockout mice. After anesthesia, the heart rate was recorded at the baseline level. Normal saline (NS) was intraperitoneally injected as the vehicle. After 15 min of recording, atropine was intraperitoneally injected at the dose of 3 mg/kg. After the injection, the heart rate was monitored continuously and the average heart rate was recorded every 5 min. The details of the experimental timeline are shown in supplementary material (**Figure S1A**). There was no significant difference in baseline heart rate between Na_V_1.8 knockout mice and wild type mice. After atropine treatment, the heart rate in wild type mice exhibited a significantly increased trend, while the heart rate response to atropine in Na_V_1.8 knockout mice did not exhibit obvious change. To make the heart rate data of different mice comparable, we normalized the heart rate after treatments by dividing each mouse’s basal heart rate. There was a significant decreased in each time points after atropine treatment in Na_V_1.8 knockout mice compared to wild type mice (5 min after atropine treatment, 1.016 vs. 1.239, *P*<0.001; 10 min, 1.019 vs. 1.235, *P*<0.001; 15 min, 1.024 vs. 1.213, *P*<0.01; 20 min, 1.03 vs. 1.221, *P*<0.01; 25 min, 1.019 vs. 1.209, *P*<0.01; 30 min, 1.027 vs. 1.201, *P*<0.01; 35 min, 1.03 vs. 1.211, *P*<0.01; 40 min, 1.019 vs. 1.209, *P*<0.01; 45 min, 1.03 vs. 1.214, *P*<0.05) (**Figures 4A, B**).

**Figure 5:**
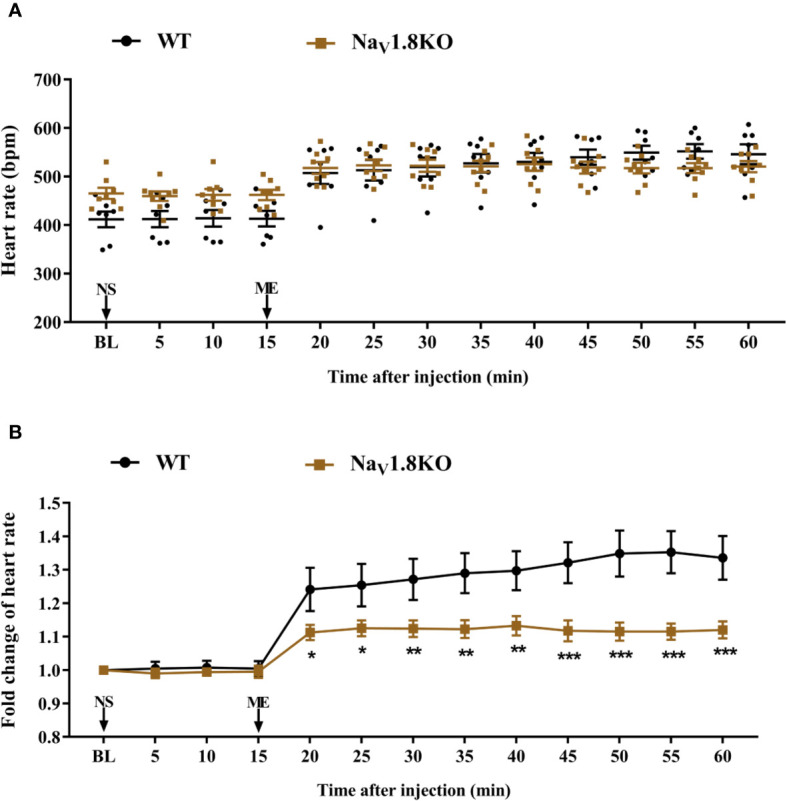
The heart rate response of methoctramine in Na_V_1.8 knockout mice. **(A)** Scatter diagram of heart rate after methoctramine application. **(B)** Line diagram of fold change of heart rate after methoctramine treatment. Heart rate was normalized to the value of baseline heart rate for each group. *p < 0.05, **p < 0.01, ***p < 0.001 vs wild type mice. WT, wild type; KO, knock out; NS, normal saline; ME, methoctramine; BL, baseline.

The cardiac effect of atropine is achieved by antagonizing the myocardial muscarinic acetylcholine receptor M2. To further explore the underlying mechanism of decreased heart rate response to atropine in Na_V_1.8 knockout mice, we examined the heart rate before and after methoctramine treatment in Na_V_1.8 knockout mice. Methoctramine is an M2-selective competitive antagonist of muscarinic acetylcholine receptors (Eglen and Watson, 1996; Jakubik et al., 2014). After anesthesia, the heart rate was recorded at the baseline level. NS was intraperitoneally injected as the vehicle. After 15 min of recording, methoctramine was intraperitoneally injected at a dose of 3 mg/kg. After the injection, the heart rate was monitored continuously and the average heart rate was recorded every 5 min (**Figure S1B**). The heart rate in wild type mice increased significantly after methoctramine treatment. After methoctramine treatment, the heart rate in wild type mice performed an obviously increased trend. The heart rate response to methoctramine in Na_V_1.8 knockout mice exhibit mitigatory change. The heart rate response to methoctramine in Na_V_1.8 knockout mice was significantly decreased compared to wild type mice (5 min after methoctramine treatment, 1.113 vs. 1.241, *P*<0.05; 10 min, 1.125 vs. 1.254, *P*<0.05; 15 min, 1.124 vs. 1.271, *P*<0.01; 20 min, 1.123 vs. 1.29, *P*<0.01; 25 min, 1.133 vs. 1.297, *P*<0.01; 30 min, 1.118 vs. 1.321, *P*<0.001; 35 min, 1.115 vs. 1.349, *P*<0.001; 40 min, 1.115 vs. 1.353, *P*<0.001; 45 min, 1.12 vs. 1.336, *P*<0.001) (**Figures 5A, B**).

**Figure 6:**
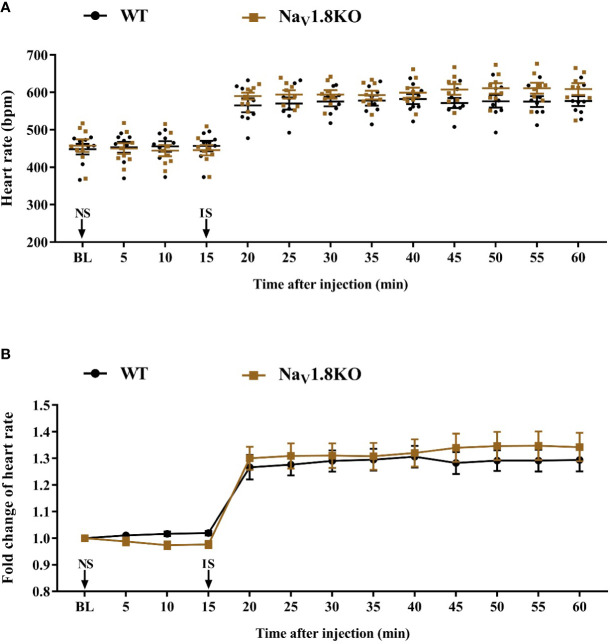
The heart rate response of isoprenaline in Na_V_1.8 knockout mice. **(A)** Scatter diagram of heart rate after isoprenaline application. **(B)** Line diagram of fold change of heart rate after isoprenaline treatment. Heart rate was normalized to the value of baseline heart rate for each group. WT, wild type; KO, knock out; NS, normal saline; IS, isoprenaline; BL, baseline.

To exclude the correlation between Na_V_1.8 and the cardiac adrenergic receptor, and to exclude a general impairment of maximum heart rate in Nav1.8 knockout mice, we examined the heart rate before and after isoprenaline treatment in Na_V_1.8 knockout mice. After anesthesia, the heart rate was recorded at the baseline level. NS was intraperitoneally injected as the vehicle. After 15 min of recording, isoprenaline was intraperitoneally injected at the dose of 2 mg/kg. After the injection, the heart rate was monitored continuously and the average heart rate was recorded every 5 min (**Figure S1C**). There was no significant difference in baseline heart rate between Na_V_1.8 knockout mice and wild type mice. The heart rates in both wild type mice and Na_V_1.8 knockout mice increased significantly after isoprenaline treatment (**Figures 6A, B**).

**Figure 7:**
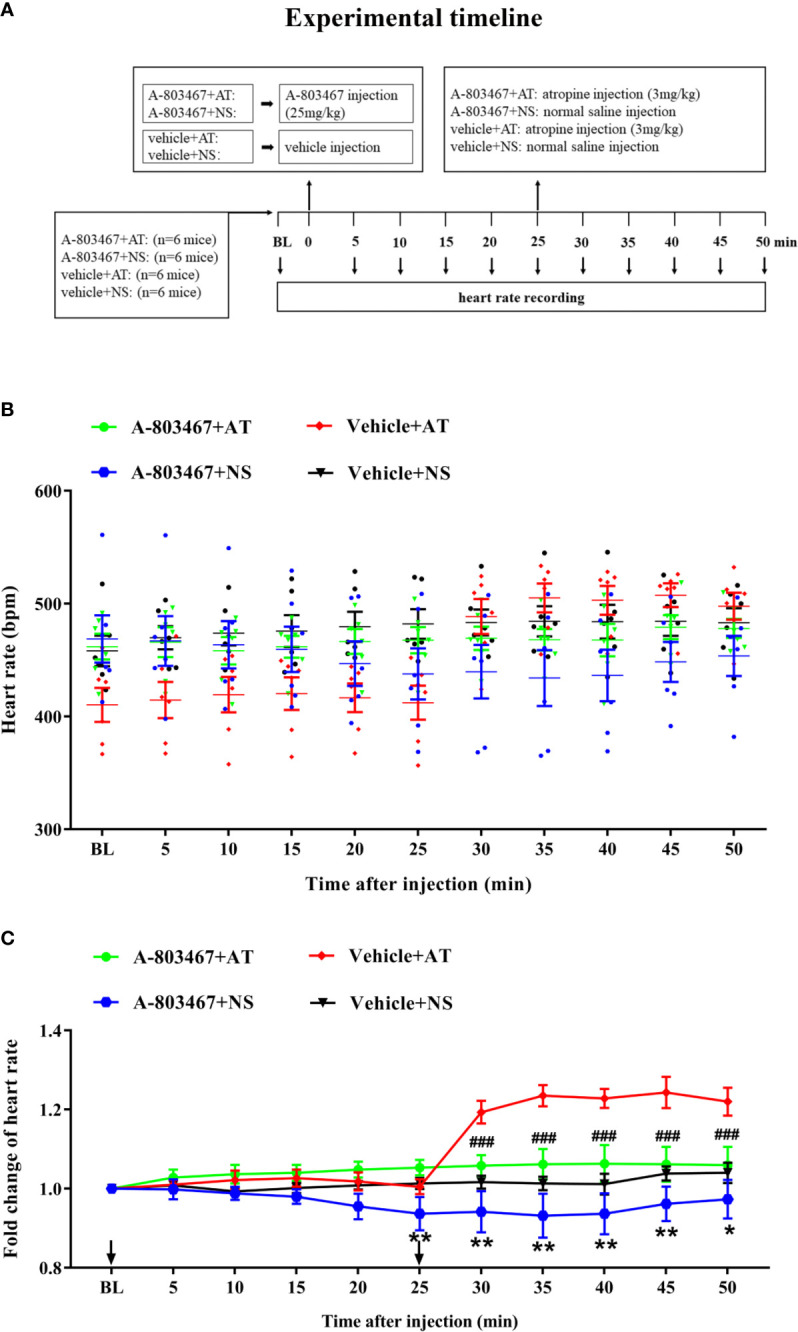
The effect of Na_V_1.8 inhibitor in heart rate response of atropine in wild type mice. **(A)** Timeline of the experiment. **(B)** Scatter diagram of heart rate after A-803467 and atropine treatment. **(C)** Line diagram of fold change of heart rate after A-803467 and atropine treatment. Heart rate was normalized to the value of baseline heart rate for each group. *means *p* < 0.05, **means *p* < 0.01 vs vehicle+NS group. ^###^means *p* < 0.001 vs vehicle + atropine group.

To further confirm the correlation between Na_V_1.8 and heart rate response to atropine, the Na_V_1.8 selective inhibitor A-803467 was intraperitoneally injected before drug treatment in wild type mice. After anesthesia, the heart rate was recorded at the baseline level. A-803467 was intraperitoneally injected at the dose of 25 mg/kg. After 25 min of recording, atropine (3 mg/kg) was intraperitoneally injected. After the injection, the heart rate was monitored continuously and the average heart rate was recorded every 5 min (**Figure 7A**). The heart rate in the A-803467+NS group was significantly decreased compared to the vehicle+NS group (25 min after A-803467 treatment, 0.9367 vs. 1.013, *P*<0.01; 30 min, 0.9417 vs. 1.017, *P*<0.01; 35 min, 0.9317 vs. 1.013, *P*<0.01; 40 min, 0.9367 vs. 1.012, *P*<0.01; 45 min, 09617 vs. 1.038, *P*<0.01; 50 min, 0.9733 vs. 1.04, *P*<0.05). The hearts rate response to atropine in the A-803467+atropine group was obviously decreased compared to the vehicle+atropine group (30 min after atropine treatment, 1.058 vs. 1.193, *P*<0.001; 35 min, 1.062 vs. 1.232, *P*<0.001; 40 min, 1.063 vs. 1.228, *P*<0.001; 45 min, 1.062 vs. 1.243, *P*<0.001; 50 min, 1.06 vs. 1.22, *P*<0.001). (**Figures 7B, C**).

## Discussion

This study indicated that a patient’s response to atropine may be affected by different *SCN10A* rs6795970 alleles (G>A; p.Ala1073Val). The allele (AA), which induces the loss-of-function of Na_V_1.8 (Duan et al., 2016b), was associated with a weaker response to atropine. Additionally, the association between loss-of-function of Na_V_1.8 and weaker response to atropine was further validated in a mice model using Na_V_1.8-knockout mice and a Na_V_1.8 inhibitor. Thus, combined with the current genetic data, our study indicated that the loss-of-function of Na_V_1.8 may decrease the heart rate in response to atropine.

*SCN10A*/Na_V_1.8 is correlated with cardiac conduction and arrhythmia (Chambers et al., 2010; Di Stolfo et al., 2018; Macri et al., 2018; Odening, 2019). Several SNPs of *SCN10A*, such as rs10428132, rs6795970, and rs6599251, are associated with PR interval and QRS duration (Chambers et al., 2010; Holm et al., 2010; Iio et al., 2015; Macri et al., 2018). In addition, Qi et al. showed that the Na_V_1.8 blocker A-803467 significantly eliminates cervical vagus nerve stimulation-induced conduction and atrial fibrillation inducibility (Qi et al., 2014). In the present study, the *SCN10A* variant rs6795970 was associated with heart rate response to atropine in the human population. There was no significant difference in basal heart rate among people with different *SCN10A* rs6795970 alleles. However, after atropine treatment, the heart rate response in patients with AA was significantly lower than those in patients with GG and GA. This result was observed in unmatched and matched populations (electrocardiographic data including PR interval, QRS duration, and QT interval were also included as matched factors). Our results revealed the correlation between *SCN10A* variant rs6795970 and heart rate response to atropine. Several studies have demonstrated that rs6795970 accelerates inactivation and decreases persistent currents of Na_V_1.8 (Behr et al., 2015; Jabbari et al., 2015; Duan et al., 2016b). Therefore, we speculated that the decreased heart rate response to atropine in patients with variant rs6795970 may contribute to the dysfunction of Na_V_1.8. However, the cellular mechanisms by which the rs6795970 variant produces the altered response to atropine on heart rate need further investigate.

In our results, after atropine treatment, the heart rate in wild type mice significantly increased, while the heart rate response to atropine in Na_V_1.8 knockout mice were significantly lower than that in wild type mice. It suggested that Na_V_1.8 played an important role in the cardiac response to atropine. Most intrinsic cardiac neurons in cardiac ganglionated plexi are cholinergic and the function of parasympathetic components is dominant (Hoover et al., 2009). Moreover, a study by Verkerk et al. showed that most Na_V_1.8 positive neurons in isolated neurons from murine intracardiac ganglia are cholinergic in origin (Verkerk et al., 2012). The heart rate response was significantly lower in Na_V_1.8 knockout mice than in wild type mice after the antagonist of cardiac muscarinic acetylcholine receptor M2, methoctramine treatment. these In addition, our results showed that the heart rate in both wild type mice and Na_V_1.8 knockout mice increased significantly after isoprenaline treatment. Taken together, it indicated that cardiac muscarinic acetylcholine receptor M2 may involve in the association between Na_V_1.8 and cardiac conduction. After Nav1.8 blocker A-803467 treatment, basal heart rate in wild type mice deceased. A previous study also showed that A-803467 treatment results in the prolongation of PR and QRS intervals (Sotoodehnia et al., 2010). This suggests that the blockade of Na_v_1.8 may result in a decrease in cardiac conduction and heart rate. In our study, the heart rate response to atropine was lower in Na_V_1.8 knockout mice and after Na_V_1.8 blocker A-803467 treatment in wild type mice than in wild type mice. These results demonstrated that Na_V_1.8 is involved in the atropine-induced cardiac effect. The blockade of Na_V_1.8 may result in the inhibition of cardiac neural activity and subsequently affect cardiac conduction. This indicates that Na_V_1.8 plays a critical role in cardiac electrophysiology. Therefore, blockers of Na_V_1.8, such as A-803467, may be used as a candidate therapeutic strategy for the management of cases of hyper-response to atropine during the perioperative period.

As mentioned above, the expression of Nav1.8 in heart is rather controversial. Therefore, the underling mechanism of Nav1.8 in cardiac atropine response is complicated and inconclusive. After cardiac vagus nerve suppressed by blocking muscarinic receptors, the cardiac sympathetic nerve will become the preponderant control nerve and result in heart rate increase. Numbers of studies have been indicated that substantial Nav1.8 expressed in intracardiac ganglia neurons (Verkerk et al., 2012; Casini et al., 2019). Therefore, Nav1.8 may involve in the regulation of intracardiac nerves function. For example, when the cardiac vagus nerve suppressed by atropine, the dysfunction of cardiac sympathetic may cause failure of increased heart rate response. On the other hand, it has been found that Nav1.8 was present in cardiomyocytes (Facer et al., 2011). So, we speculated that Nav1.8 may also play a role in electrophysiology function of cardiomyocytes. The dysfunction of Nav1.8 may impact the action potential generation and electrophysiological function of cardiomyocytes. Therefore, after muscarinic receptors were blocked by atropine, the dysfunction of electrophysiology of the cardiomyocytes may result in failure of increased heart rate response. Through our results, we validated that the association between Nav1.8 sodium channels and cardiac atropine response. However, the signal pathway between Nav1.8 sodium channels and cardiac muscarinic receptors need further investigated.

There was no significant difference in basal heart rate among different genotypes of rs6795970 and between Na_V_1.8 knockout mice and wild type mice. Similarly, Chamber et al. showed that, except for a shorter PR interval, the heart rate and other electrocardiogram parameters in *Scn10a*^-/-^ mice are not significantly different than those of their wild type littermates (Chambers et al., 2010). In addition, Stroud et al. showed that the heart rate in *Scn10a*^-/-^ mice is not significantly different than that in their wild type littermates (Stroud et al., 2016). As mentioned above, after A-803467 treatment, basal heart rate in wild type mice deceased. One of the causes of these contradictory results can be speculated by the difference of acute and chronic loss of Na_V_1.8. Na_V_1.8 knockout mice may develop tolerance and compensation due to chronic deficiency of Na_V_1.8. For example, neurotransmitters, such as dopamine, norepinephrine, and epinephrine, may modulate heart rate by acting on the myocytes or Na_V_1.8 negative neurons. However, since lack of isolated cell models, the cause of the contradictory result of no difference in basal heart rate of Na_V_1.8 null mice with that Na_V_1.8 antagonist decrease basal heart rate is hard to explain. The detailed mechanism behind contradictory results need to further investigate.

However, this study had some limitations. First, the clinical study was a retrospective study of the female surgical population. To improve the generalizability of the results, the association between *SCN10A* and heart rate response to atropine needs to be verified by a prospective clinical study with a larger sample of both male and female populations. Second, because the main purpose of this study was to provide evidence for the link between Na_V_1.8 and the heart rate response to atropine, we examined the heart rate change before and after drug treatment and did not use the patch clamp technique to verify the change in electrophysiology parameters in cardiomyocytes. Third, future study is needed to explore the specific cellular mechanism underlying the effect of Nav1.8 on heart rate response to atropine. Nevertheless, this study expands the knowledge of the role of Na_V_1.8 in the cardiac pharmacological response. Atropine is one of the most commonly used drugs for heart rate regulation and is used frequently in clinical practice, such as during the perioperative period and in intensive care units. The significant individual differences in the response to atropine remain a clinical problem and it is still difficult to explain and manage heart rate in the clinical context. This study validates the role of Na_V_1.8 in the cardiac response to atropine in human and animal models.

This study expanded the potential function of Na_V_1.8 in the heart rate response to atropine using evidence in humans and *in vivo*. In addition, we speculated that Na_V_1.8 may regulate the cardiac response to atropine by modulating the cardiac muscarinic acetylcholine receptor M2. It may provide new ideas and intervention targets for the pharmacological research of clinical heart rhythm regulating drugs.

## Data Availability Statement

The original contributions presented in the study are included in the article/**Supplementary Material**; further inquiries can be directed to the corresponding authors.

## Ethics Statement

The study was a retrospective study and the protocol was approved by the Institutional Ethics Committee of Tongji Hospital, Tongji Medical College, Huazhong University of Science and Technology. The cohort was obtained from our previous study (Duan et al., 2016a) and, because this study was based on the previous data, written informed consent was waived.

## Author Contributions

GD and XZ designed the study. NL, MZ, and YL analyzed and interpreted the patient data. BL, JZ, WW, and YH performed the animal experiments. BL and NL analyzed data. BL and GD prepared the manuscript. All authors contributed to the article and approved the submitted version.

## Funding

This work was supported by National Natural Science Foundation of China (NO.81701096).

## Conflict of Interest

The authors declare that the research was conducted in the absence of any commercial or financial relationships that could be construed as a potential conflict of interest.
