# Supplementary figures and images for: The Role of Voltage-Gated Sodium Channel 1.8 in the Effect of Atropine on Heart Rate: Evidence From a Retrospective Clinical Study and Mouse Model
Source: Front Pharmacol. 2020 Jul 31;11:1163. doi: 10.3389/fphar.2020.01163 (PMC7412993; doi:10.3389/fphar.2020.01163)

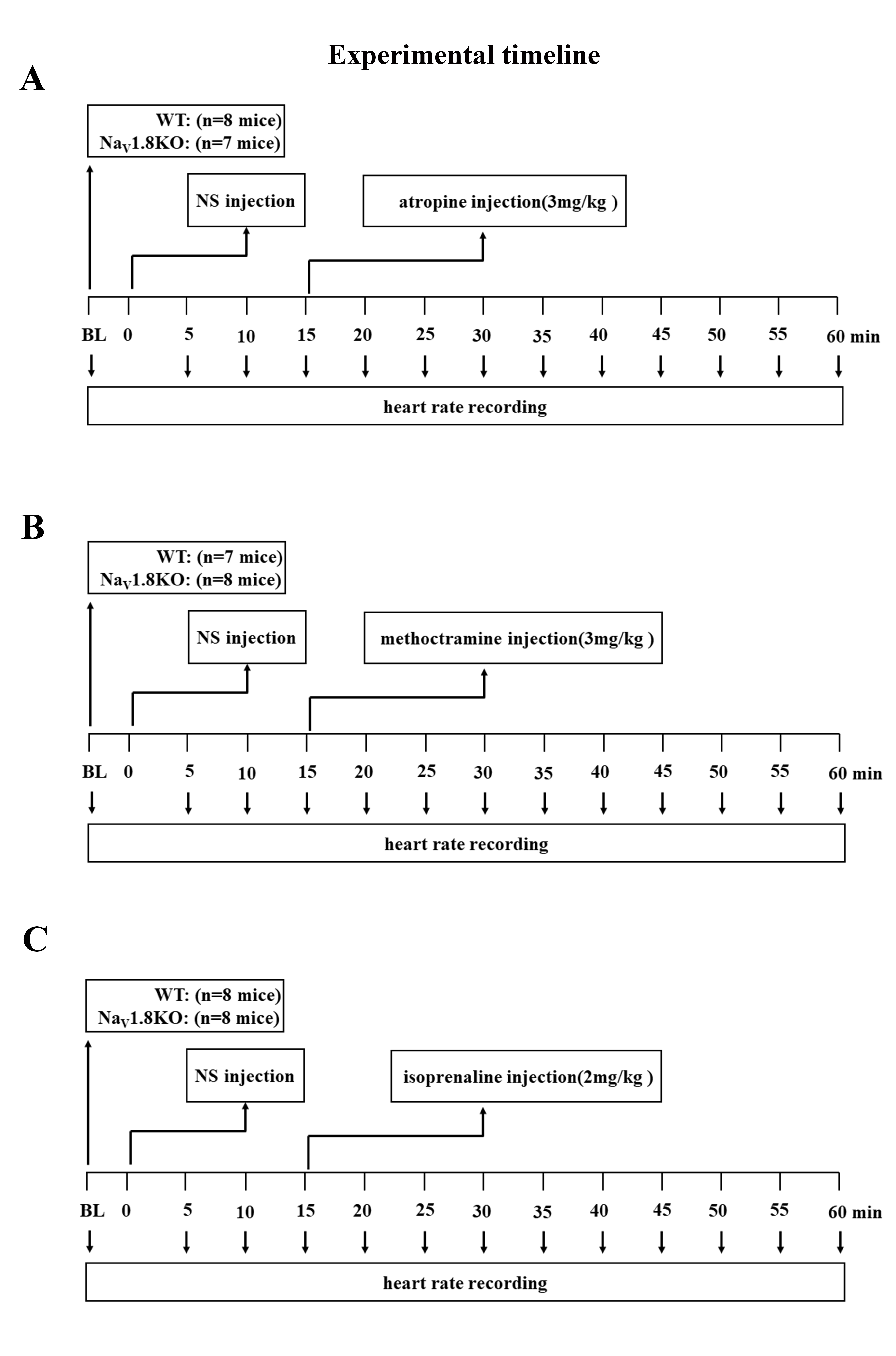

Supplement: Supplementary file 2 [file Image_1.jpeg]
